# Single-base resolution methylome analysis shows epigenetic changes in *Arabidopsis* seedlings exposed to microgravity spaceflight conditions on board the SJ-10 recoverable satellite

**DOI:** 10.1038/s41526-018-0046-z

**Published:** 2018-07-12

**Authors:** Peipei Xu, Haiying Chen, Jing Jin, Weiming Cai

**Affiliations:** Laboratory of Photosynthesis and Environment, CAS Center for Excellence in Molecular Plant Sciences, Shanghai Institute of Plant Physiology and Ecology, Chinese Academy of Sciences, No. 300 Fenglin Road, Shanghai, 200032 China

## Abstract

DNA methylation is a very important epigenetic modification that participates in many biological functions. Although many studies of DNA methylation have been reported in various plant species, few studies have assessed the global DNA methylation pattern in plants challenged by exposure to microgravity conditions. In this report, we mapped the *Arabidopsis* genome methylation pattern changes associated with microgravity conditions on board the Chinese recoverable scientific satellite SJ-10 at single-base resolution. Interestingly, we found epigenetic differences in *Arabidopsis* seedlings exposed to microgravity in that the *Arabidopsis* genome exhibits lower methylation levels in the CHG, CHH, and CpG contexts under microgravity conditions. Microgravity stimulation was related to altered methylation of a number of genes, including DNA methylation-associated genes, hormone signaling related genes, cell-wall modification genes and transposable elements (TEs). Relatively unstable DNA methylation of TEs was responsible for the induction of active transposons. These observations suggest that DNA demethylation within TEs may affect the transcription of transposons in response to microgravity conditions. In summary, the results of this investigation are beneficial for understanding the mechanism of plant adaptation to microgravity and improve strategies to allow plants to adapt to space.

## Plant biology: Spaceflight leaves its epigenetic mark on seedling DNA

Spaceflight alters the pattern of chemical tags that adorn DNA in plant seedlings. Weiming Cai and colleagues from the Shanghai Institute of Plant Physiology and Ecology, China, profiled the genome-wide epigenetic patterns of *Arabidopsis thaliana* seedlings that spent 60 h in the microgravity of low Earth orbit aboard the Shijian-10 recoverable satellite. They analyzed the distribution of methyl tags across the genome — an epigenetic mark that affects gene expression levels — and found that seedlings exposed to microgravity had lower methylation on average than control plants grown on the ground, although certain genes related to methylation, transcription factors and hormones tended to be more methylated. Epigenetic differences were also observed among genes involved in cell-wall modification and in transposable elements. The findings could help inform the design of plants optimized for growth in space.

## Introduction

On Earth, life is adapted to a constant gravitational force, and biological processes in organisms have evolved under this natural constant. Many limiting factors for plant growth under microgravity conditions have been identified. Therefore, it is expected that plants have sufficient and sustainable mechanisms that allow for them to survive under microgravity conditions. To identify plant responses to the microgravity stimulus, many groups have been using various approaches to identify genes with altered expression levels under microgravity conditions.^1–5^ The results have shown that microgravity signals can be transduced into molecular signaling cascades, which lead to plant adaptation.

Currently, it is obvious that epigenetic alterations are involved in plant adaptation to environmental stress.^6^ A series of current reports have shown that DNA methylation leads to control of gene mRNA levels and plays important roles in abiotic and biotic stresses.^6^ For example, drought stress results in a global DNA methylation alteration in rice.^7^ Salt stress induces *OsMYB91* gene expression due to the reduced cytosine methylation level in its promoter region.^8^ In addition, widespread alterations in DNA methylation of the poplar genome in response to drought treatment indicate adaption to the local environment.^9^ Moreover, epigenetic variations in transposable element (TE) regions can be affected by environmental stresses.^10^ Silencing of the *ZmMET1* gene after low-temperature treatment leads to demethylation of the Ac/Ds transposon region in maize roots.^11^ In sum, these results indicate an association between environmental stresses and DNA methylation alterations.

DNA cytosine methylation functions as an important controller of gene mRNA levels. In the *Arabidopsis* genome, DNA cytosine methylation is mostly detected at CpG sites and can also occur at CHG and CHH residues (H indicates A, T, or C).^12^ In the *Arabidopsis* genome, most methylation in the gene body is detected at CpG sites, while CpG, CHH, and CHG site methylation occurs elsewhere and in repetitive regions.^12^ CG methylation is enriched in TEs and gene bodies, but non-CG methylation is mostly present in TEs.^13^ Often, methylation of gene promoter regions is believed to inhibit gene expression, while methylation within introns and exons can promote gene transcription.^14^ MET1 is the main CG methyltransferase, CMT3 is the main CHG methyltransferase, and DRM2 is the main CHH methyltransferase and is guided by small RNAs.

Epigenetic features play a critical role in controlling gene expression and the subsequent response of an organism to its environment. As a major epigenetic modification, DNA methylation is not directly encoded in the genome sequence, and yet can modify expression and may be inherited for at least one generation. Several studies have shown that large numbers of plant genes are differentially expressed in response to spaceflight.^4^ Learning more about the spaceflight methylome of plants will contribute to the foundational understanding of how plants adapt to spaceflight. In this research, we showed the results of an SJ-10 spaceflight experiment. The experiment was a part of the SJ-10 mission, an experimental project of Chinese Academy of Sciences in April 2016. *Arabidopsis* seedlings were exposed to spaceflight on board of SJ-10. To evaluate DNA methylome during spaceflight, we profiled DNA methylation genome wide in *Arabidopsis* seedlings. Whole-genome bisulfite sequencing (BS-Seq) allows DNA methylation to be measured at the whole-genome level with single nucleotide resolution.^13^ These results will be very useful for our understanding of the potential role of cytosine DNA methylation in plants adapting to the outer space environment.

## Results

### Experimental design and sample fixation on the recoverable scientific satellite SJ-10

**Fig. 1:**
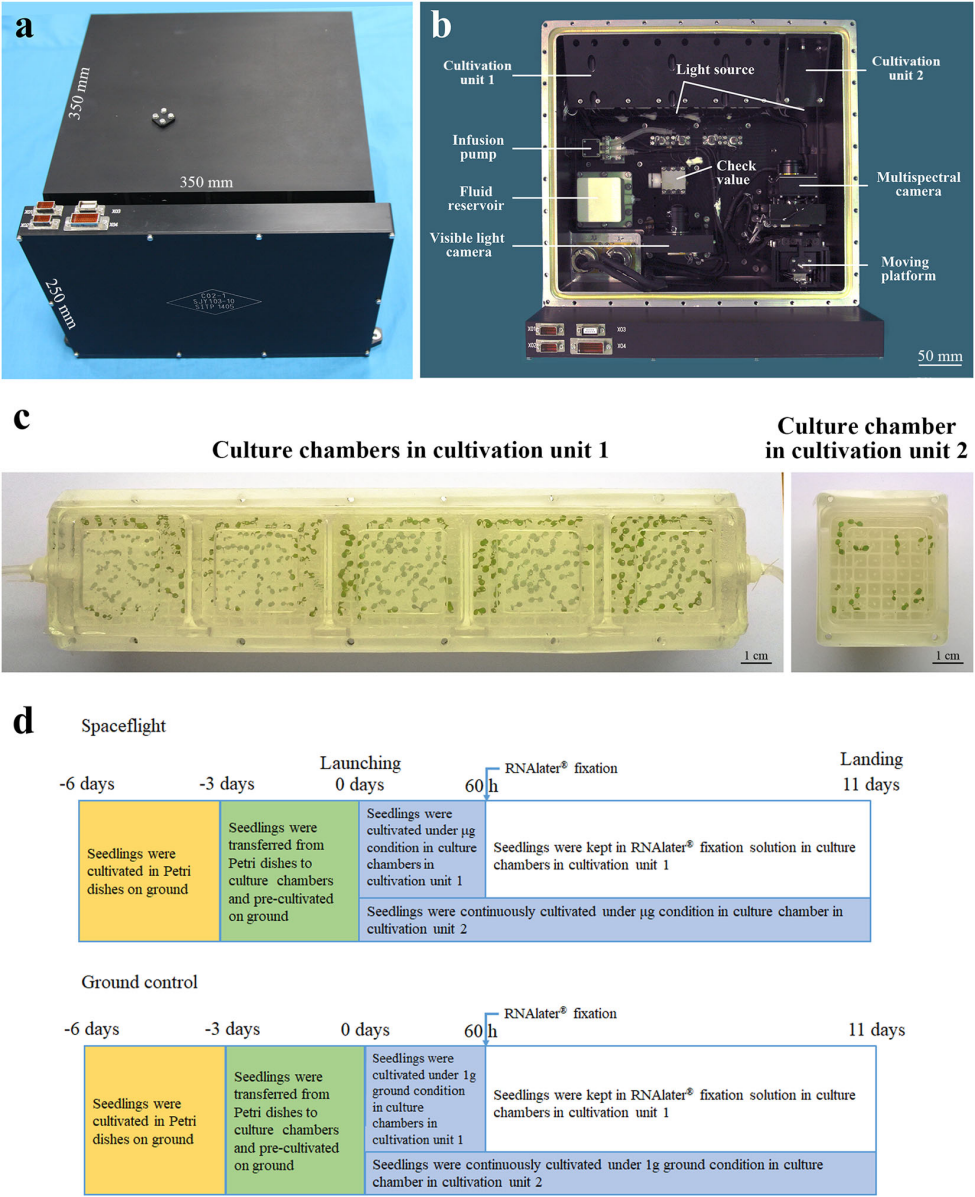
Hardware and experimental procedures used in the experiment on board the SJ-10 recoverable satellite. **a** Appearance of the hardware used in the spaceflight experiment. The equipment functioned as a housing for the culture chambers. **b** Distribution of internal components of the hardware. The hardware includes culture chambers, a pump support system, a canal system and a fixative unit connected to the culture chambers. **c** *Arabidopsis* seedlings were grown in culture chambers during the space experiment. **d** Schematic view of the *Arabidopsis* seedlings on board the SJ-10 satellite during spaceflight and on the ground. *Arabidopsis* seedlings in culture chambers were transferred from Petri dishes 3 days before launching. Seedlings in culture chambers installed in cultivation unit 1 were grown for 60 h under microgravity conditions and fixed in space using RNAlater®. The seedlings in the culture chamber installed in cultivation unit two grew for 11 days under microgravity; after this time, they were still alive and returned with the satellite. The g-profile during SJ-10 satellite in orbit is described in the references.^15,16^ The g-profile during launch was as follows: the first-level maximum static overload was 4.8 g in flight for 150 s; the second-level maximum static overload was 6.0 g in flight for 180 s

The specific equipment used in the space experiment was designed and constructed by Shanghai Institution of Technical Physics (Fig. 1a, b). The equipment included two cultivation units, a canal system and a pump support system. The polysulfone chambers in the cultivation units had windows that were covered by a gas permeable membrane. The chambers were connected to the fixative unit by tube connectors. The Chinese recoverable scientific satellite SJ-10 was launched at 01:38 on April 6, 2016 and landed at 16:30 on April 18, 2016. The air temperature near the incubator averaged approximately 23 °C. The average relative humidity near the incubator was approximately 25%, and the microgravity level was estimated to be approximately 10^−3^ to 10^−4^g during the unified flight phase.^15,16^ Seedlings were fixed with RNAlater® after growth for 60 h under microgravity conditions. They were harvested approximately 4 h after landing, stored at 4 °C, and transported to our laboratory for further assays. Seedling DNA was extracted and used for methylome analysis. To halt cellular activities in space and preserve the DNA methylome profile, RNAlater® was added to the culture chambers on board SJ-10 and on the ground. RNAlater® has been experimentally verified to effectively preserve DNA and to yield high-quality RNA and has been used successfully in spaceflight applications.^17^ Samples of the 1 g ground controls in cultivation unit 1 were also fixed in RNAlater® at the same time as the in-space samples (Fig. 1d).

### Global DNA methylation patterns of *Arabidopsis* seedlings under microgravity conditions

**Fig. 2:**
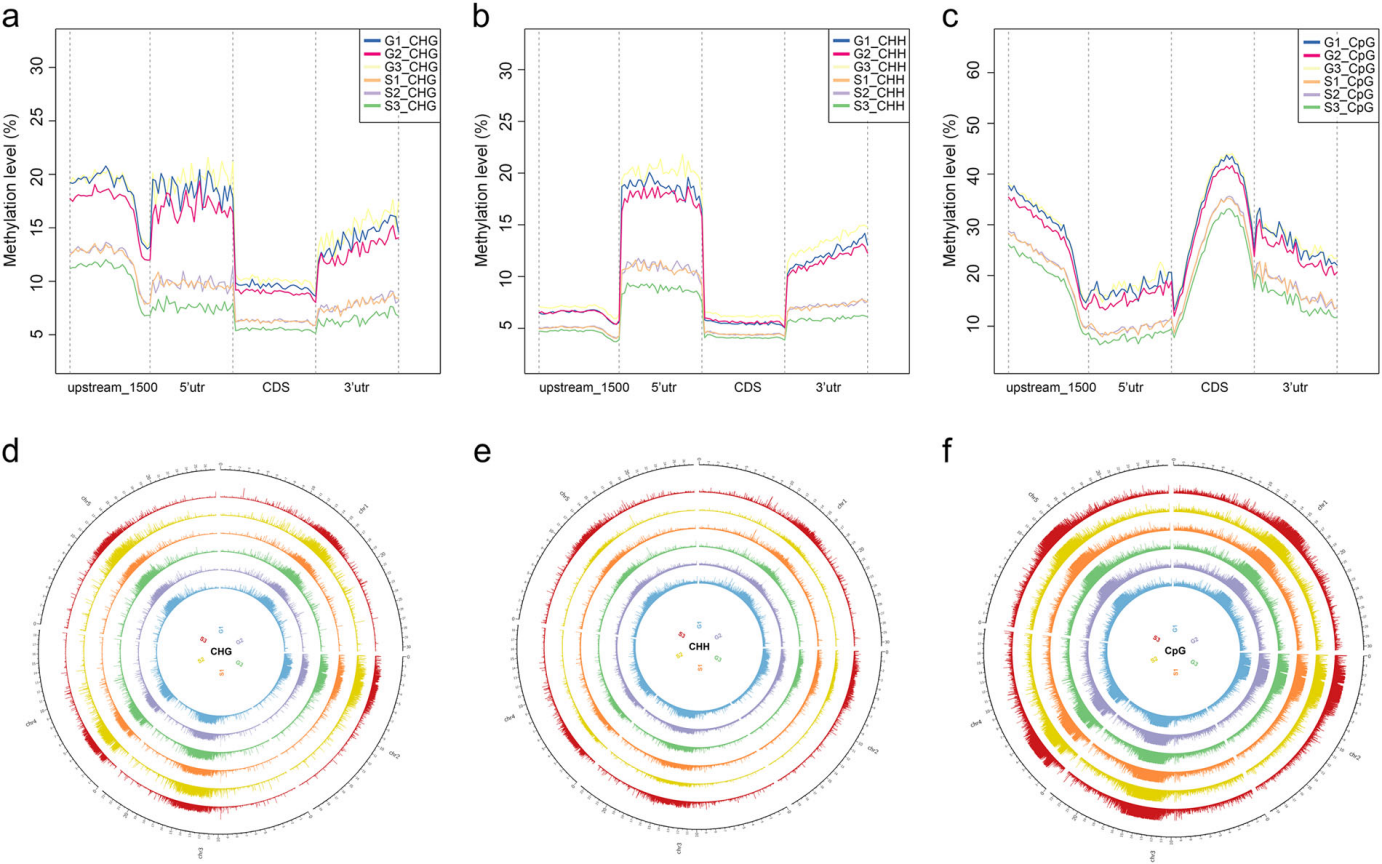
DNA methylation patterns in various genomic regions. **a–c** Distribution of the DNA methylation levels in the CHG **a**, CHH **b**, and CpG **c** contexts among various gene regions, including the promoter, 5’UTR, gene body and 3’UTR in ground controls (G1–G3), and in plants maintained under microgravity conditions (S1–S3). **d**–**f** Circos plots showing *Arabidopsis* genomic methylation in ground controls (G1–G3) and in plants maintained under microgravity conditions (S1–S3). The outer track represents five chromosomes of the *Arabidopsis* genome. The other histogram tracks represent differentially methylated regions in the CHG **d**, CHH **e**, and CpG **f** contexts

To identify the DNA methylation pattern that was present in the seedlings during spaceflight, single-base resolution methylome analysis was conducted using *Arabidopsis* (Col-0) seedlings that had been grown for 60 h under microgravity conditions. The DNA methylation profiles at the whole-genome level were determined; analysis of the read intensity around the annotated genes revealed lower DNA methylation levels under microgravity conditions (Fig. 2). Because euchromatin and heterochromatic genomic regions such as coding sequences and repetitive sequence regions display different DNA methylation patterns, we further evaluated the detailed methylation patterns within genes, including coding sequences (CDS) and noncoding areas. The results showed that CpG sites had the highest methylation levels and that CHH sites had the lowest methylation levels in each gene region (Fig. 2a–c). In the CpG context, CpG methylation in the 5’UTR and 3’UTR regions was lower than that in coding regions, and the 5’UTR methylation level was much lower than that in the 1500-bp upstream region and in the 3’UTR (Fig. 2c). The CHG context appeared to be quite different from the CpG context, with much higher methylation levels in 1500-bp upstream regions and 5’UTR regions, and CDS displayed the lowest methylation level (Fig. 2a). However, the CHH context showed higher methylation levels in the 5’UTR and 3’UTR regions and lower methylation levels in the 1500-bp upstream region_-_and CDS sites (Fig. 2b). Circos plots show the differences in methylation changes between ground controls (G1–G3) and plants exposed to microgravity conditions (S1–S3) (Fig. 2d–f). The outer track indicates the five chromosomes of the *Arabidopsis* genome. The other histogram tracks represent differentially methylated regions (DMRs) in the CHG (d), CHH (e), and CpG (f) contexts.

Previous research showing that cytosine methyltransferases and demethylases play important roles in the maintenance of genomic methylation indicates that these enzymes are involved in a number of biological functions.^18^ To investigate possible altered methylation patterns in methyltransferase- and demethylase-related genes in response to microgravity conditions, we examined all of these genes to determine whether there were any changes in their DNA methylation levels under microgravity. The details of these results for *DMT1*, which encodes the cytosine methyltransferase MET1, *DME*, which encodes the DNA glycosylase DEMETER, and *TET14*, a tetraspanin family gene, are shown in Table S1. The results indicate that exposure of the plants to microgravity was accompanied by alterations in the DNA methylation patterns of specific genes.

### Altered methylation profiles of functional genes in response to microgravity conditions

**Fig. 3:**
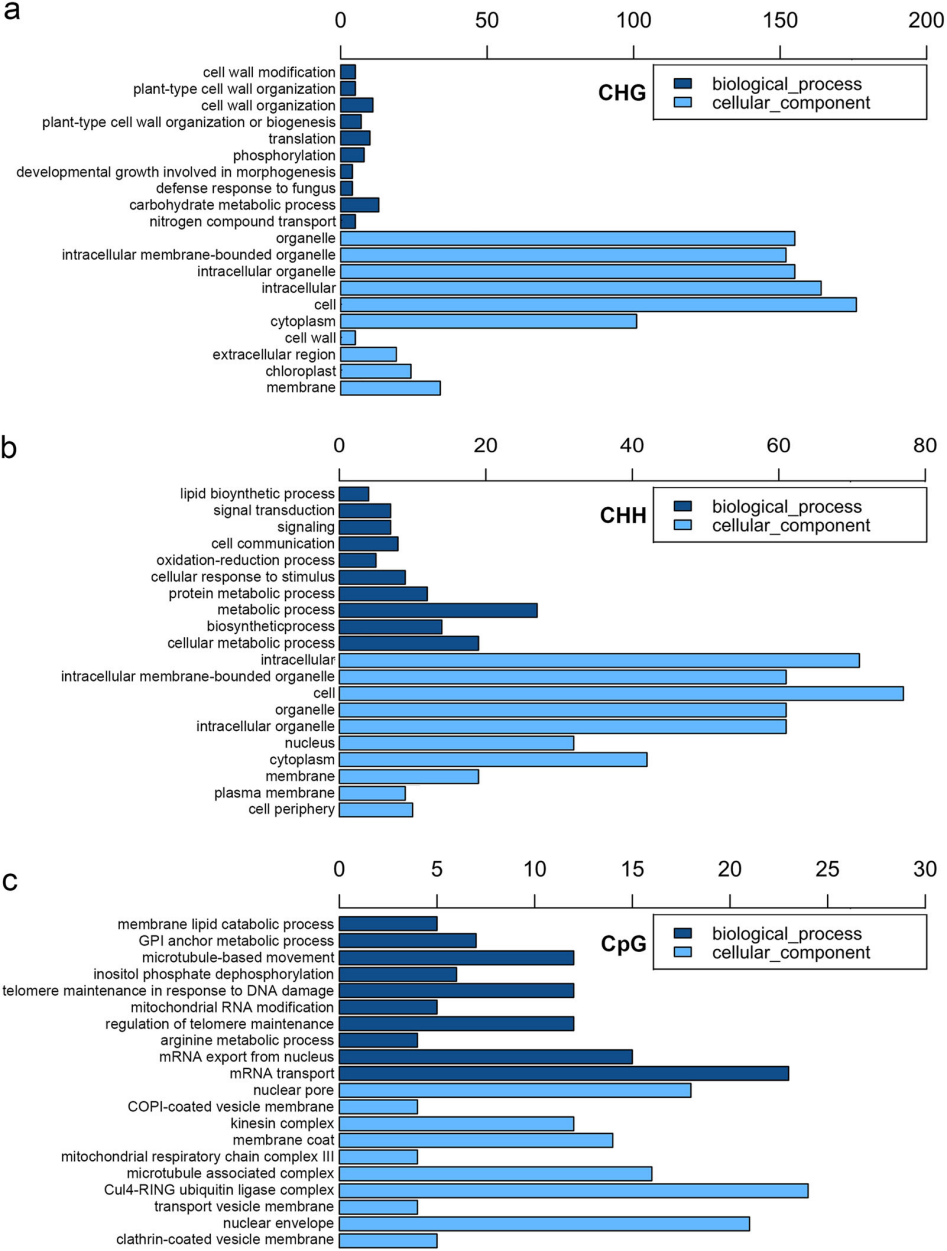
GO-term enrichment network of genes with altered methylation levels in the *Arabidopsis* genome. GO-term enrichment analysis of altered gene methylation patterns in the CHG **a**, CHH **b** and CpG **c** contexts under microgravity conditions was conducted. The bar represents the number of genes in the test set belonging to each GO category. Only the top 10 GO terms are listed (*p* < 0.05)

To perform an in-depth analysis of the functional categories of genes that showed alterations in their methylation patterns under microgravity conditions, we used gene ontology (GO) enrichment to categorize these genes. In the CHG context in the category of biological processes, altered methylation-related (AMR) genes were enriched for cell wall-related processes, carbohydrate metabolic processes, defense response, and nitrogen compound transport. Changes in these biological processes were also shown in transcriptomic studies in several previous plant spaceflight experiments.^3,4^ Although the results of various spaceflight experiments differ for a variety of reasons, changes in cell wall-related processes and defense response were consistently detected.^4^ This suggests that the weakening of the plant cell wall that occurs under microgravity may be regulated at the level of DNA methylation. Changes in metabolism-associated AMR genes, including carbohydrate metabolic processes and nitrogen compound transport, suggested that energy metabolism might be regulated in response to the microgravity environment. The defense response that occurs in plants during spaceflight might be due to the changes in forces on the cell wall under microgravity.^19^ The discovery that defense response AMR genes are expressed under microgravity may validate this assumption (Fig. 3a). In the CHH context in the category of biological processes, AMR genes for metabolic processes, cellular metabolic processes, biosynthetic processes, signaling transduction, oxidation–reduction processes, and lipid biosynthesis were enriched. Lipid biosynthesis has been implicated in gravity responses in *Arabidopsis*, and is likely involved in adjusting the polarity of cells.^20^ Here, we also found the lipid biosynthesis AMR genes. Gravity sensing in plant cells involves signals related to oxidative stress. In fact, oxidation–reduction process AMR genes were also identified. Regarding cellular components such as cell, intracellular, organelle, plasma membrane, and cell periphery, AMR genes were enriched (Fig. 3b). In the CpG context in the category of biological processes, AMR genes for mRNA transport, mRNA export from the nucleus, microtubule-based movement, telomere maintenance in response to DNA damage, regulation of telomere maintenance and inositol phosphate phosphorylation were enriched. Because space radiation can cause damage to DNA directly, the radio-adaptive response (RAR) of *Arabidopsis* root growth is modulated under microgravity conditions, and DNA damage repair in RAR is regulated by microgravity.^5^ Here, we found DNA damage and regulation of telomere maintenance AMR genes, supporting this assumption. Inositol phosphate signaling has also been shown to occur in response to microgravity conditions.^21^ For cellular components, AMR genes for the functional categories of Cul4-RING ubiquitin ligase complex, nuclear envelope, and nuclear pore were enriched (Fig. 3c).

### Numerous TE genes are hypomethylated under microgravity conditions

**Fig. 4:**
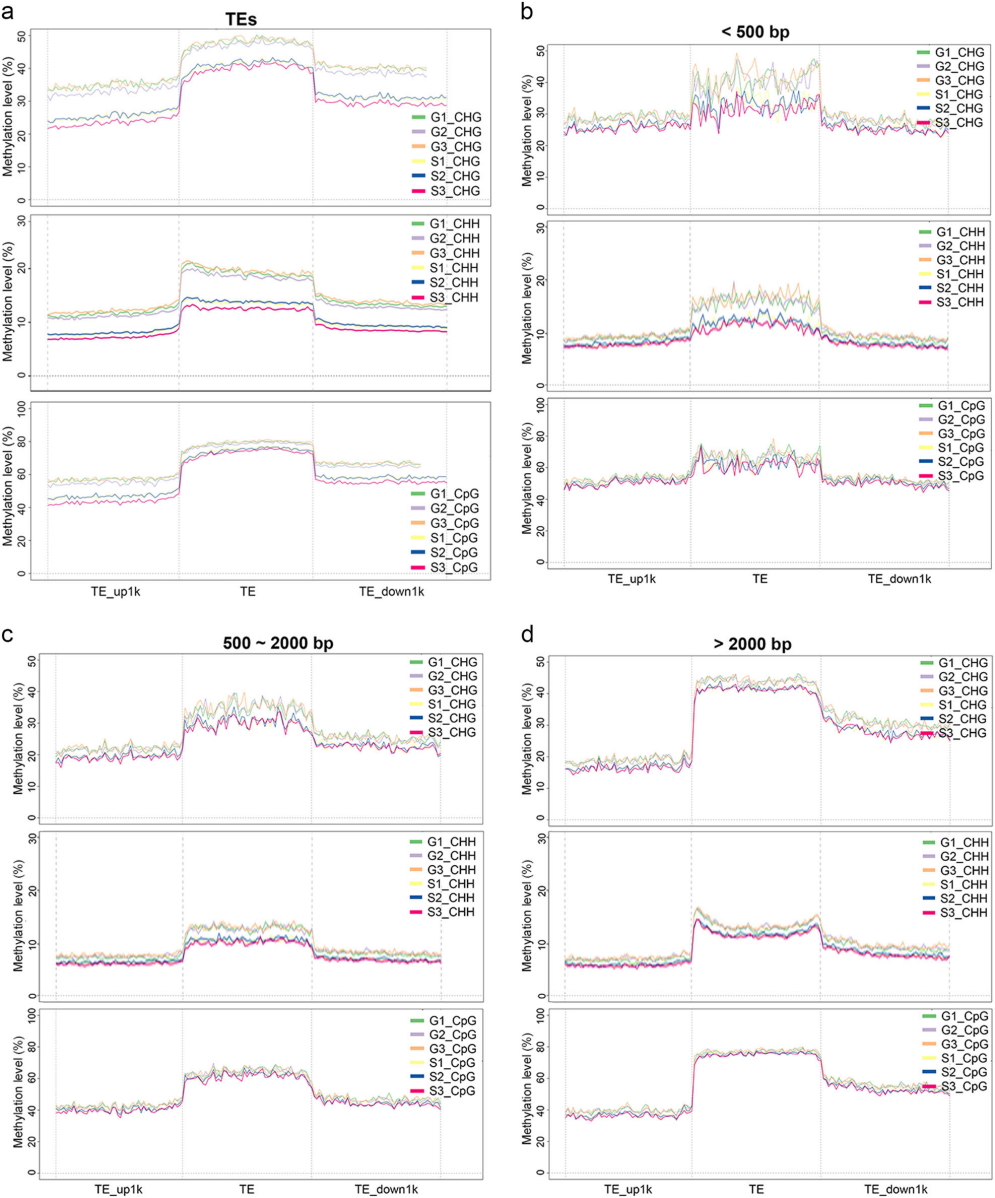
Methylation patterns of TEs and different types of TEs with different lengths in the *Arabidopsis* genome. **a** Methylation patterns of TEs. The distribution of the methylation read density for all TEs in the *Arabidopsis* genome between ground controls (G1–G3), and under microgravity conditions (S1–S3) is shown. The *x*-axis represents the TE body and its 1-kb upstream and downstream regions. The *y*-axis indicates the average methylation level. Three types of methylation patterns, CHG, CHH, and CpG, are shown in the figure. **b**–**d** Methylation patterns of TEs with different lengths. Methylation patterns of TEs with different lengths in ground controls (G1–G3) and in plants grown under microgravity conditions (S1–S3). TEs were divided into quintiles based on their lengths: the first quintile is the shortest, and the third quintile represents TEs of the longest length. The read density distribution for TEs with lengths of **(b)** less than 500 bp, **(c)** 500–2000 bp, and **(d)** more than 2000 bp within the *Arabidopsis* genome is shown. Three types of methylation patterns, CHG, CHH, and CpG, are shown in each figure

Previous research has shown that mobilization and silencing of TEs are often accompanied by disruption of DNA methylation.^22^ TEs can affect the size of the genome, create insertions and other mutations, and influence gene expression patterns. Under microgravity conditions, many differentially methylated TEs were demethylated (Fig. 4a). The methylation patterns of the CHG, CHH, CpG contexts in the TE regions and methylation levels in the 1-kb upstream and downstream regions were demonstrated (Fig. 4a). TEs presented lower methylation levels in the CHG, CHH, and CpG contexts in spaceflight samples. The results showed that a large proportion of the TEs in these contexts are differentially methylated and that hypomethylation of TE sites was likely the result of the induction of active TEs.

### Altered methylation profiles of TEs of different lengths in response to microgravity conditions

Previous research has shown that the length of TEs can affect their methylation levels.^23^ Moreover, TE methylation levels can influence transposon expression in response to environmental stress.^24^ To further identify the association between methylation status and TE length in *Arabidopsis*, TEs were divided into three groups: the first group with lengths shorter than 500 bp, second group with lengths ranging from 500 to 2000 bp; and third group with lengths of more than 2000 bp. Compared with the ground controls (G1–G3), the first group of TEs showed a striking decrease in CHG and CHH methylation levels under microgravity conditions (S1–S3) (Fig. 4b). The second group showed a marginal decrease in the CHG and CHH methylation contexts (Fig. 4c). The third group showed a decrease in the CHG context but no decrease in the CHH or CpG methylation contexts under microgravity conditions (Fig. 4d). We propose that demethylation of TEs plays an important role under microgravity conditions.

### Widespread DNA methylation in response to microgravity spaceflight

**Fig. 5:**
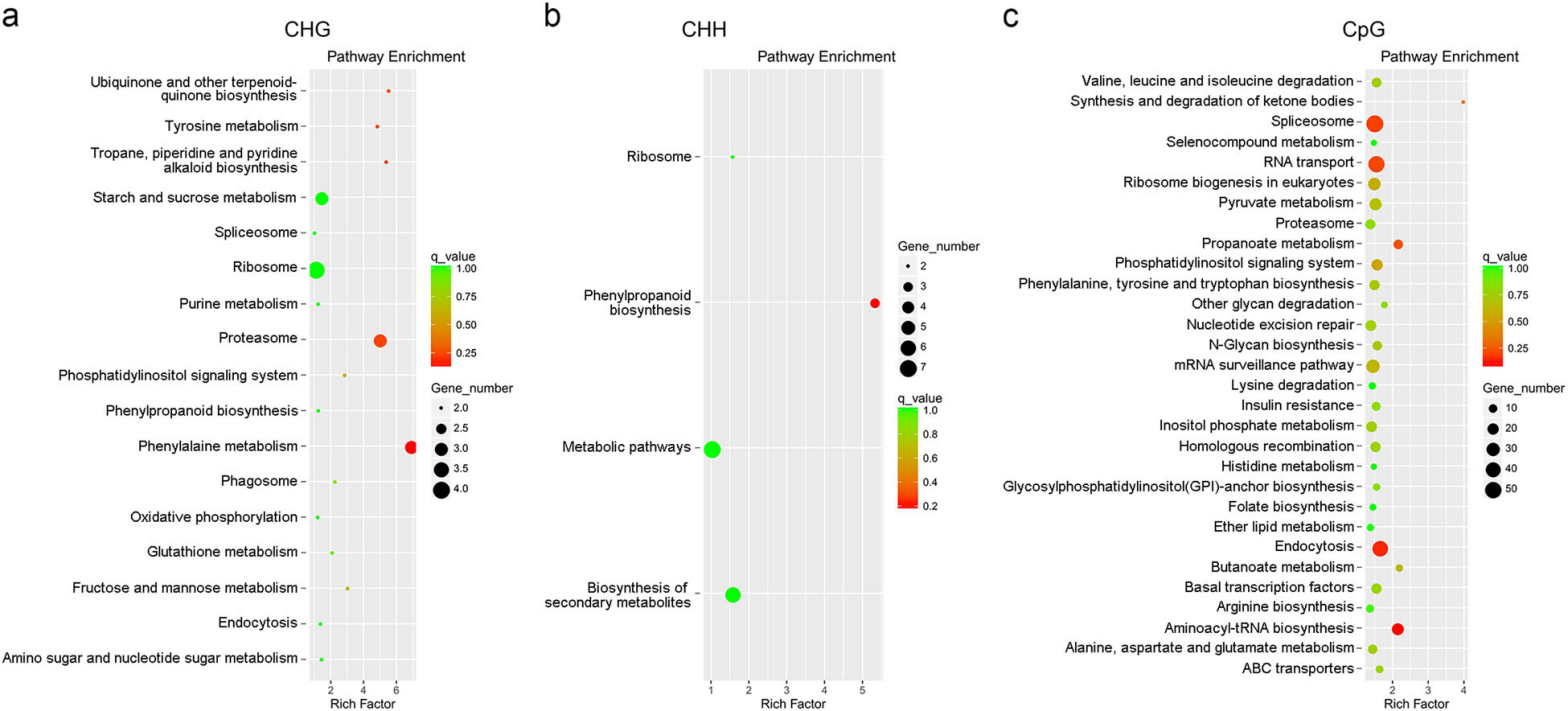
Pathway analysis of genes that showed altered methylation. KEGG pathway enrichment analysis of CHG **a**, CHH **b**, and CpG **c** differentially methylated genes in the *Arabidopsis* genome in plants grown under microgravity conditions and ground controls was performed. The size of the circle represents the gene numbers, and the color represents the *q* value

To identify the potential influence of microgravity spaceflight on the methylation level of the whole genome, we analyzed the DMRs in flight seedlings and ground controls. The distribution and methylation levels of the DMRs of five *Arabidopsis* chromosomes are shown in Figs. S3–S6. To determine the molecular functions of these DMR-related genes, we conducted KEGG pathway enrichment analysis. In the CHG context, these genes were mainly assigned to pathways associated with metabolism (starch and sucrose metabolism, phenylalanine biosynthesis, purine metabolism, glutathione metabolism, amino sugar, and nucleotide sugar metabolism), proteasomes, ribosomes, and phagosomes (Fig. 5a), whereas in the CHH context they were assigned to pathways related to metabolic pathways, biosynthesis of secondary metabolites, phenylpropanoid biosynthesis, and ribosomes (Fig. 5b). In the CpG context, they were mainly assigned to pathways associated with metabolism-related processes (such as sulfur metabolism, terpenoid backbone metabolism, N-glycan biosynthesis, butanoate metabolism, and beta-alanine metabolism), ABC transporters, nucleotide excision repair, protein procession in the ER, and endocytosis (Fig. 5c). Many of the pathways identified here in the studies of transcriptomic analysis under microgravity have also been found in other spaceflight experiments.^3,4^ Some of these pathways play important roles in plant gravity perception and in the responses of plants to microgravity. For instance, ABC transporters have been shown to play a role in the transport of the phytohormone auxin. The enrichment of AMR genes encoding ABC transporters implies that auxin transport can be modulated by microgravity conditions at the level of DNA methylation.^25^

We also further characterized methylation alterations in genes encoding transcription factors (TFs). The PlantTFDB predicts that the *Arabidopsis* genome contains more than 2200 TFs. Approximately, 36 TF genes showed altered methylation levels during microgravity spaceflight. These included bHLH, bZIP, GATA, WRKY, and NAC members (Table S3). Previous spaceflight experiments demonstrated that the expression of many TF genes is altered under microgravity.^2,3^ For example, in the TROPI-2 project, an NAC TF gene, *ANAC104/XND1* (At5g64530), was up regulated under microgravity conditions.^2^ *ANAC104/XND1* regulates the synthesis of tracheal element secondary walls and can affect plant height and the length of tracheal elements in stems and hypocotyls.^26^ Another NAC TF gene, *NAC1* (NAC domain-containing protein 21/22, At1g56010), was down regulated under microgravity.^2^ *NAC1* encodes a transcription factor that is induced by auxin and mediates auxin signaling to promote lateral root development.^27^ Alteration of the expression of auxin-related and cell wall-related genes under microgravity is closely related to plant adaptation to microgravity. Therefore, it is proposed that the possible differential methylation of genes encoding TFs may also contribute to the adaptation of plants to microgravity.

Abundant DMRs were found to be involved in the “Signaling” pathway. The observed alterations in the methylation of genes involved in hormone signaling pathways were functionally characterized. Genes involved in hormone signaling pathways, including auxin, ABA (abscisic acid), ethylene, and BRs (brassinosteroids), were also differentially methylated (Table S4). Notably, several auxin-related genes whose DNA methylation was changed under microgravity have been identified, including the genes encoding auxin responsive factors (ARF1-6, ARF8, ARF19, ARF21, and ARF22), auxin signaling F-box proteins (TIR1, AFB2, and AFB5), auxin responsive proteins (IAA9 and IAA33), transport protein (BIG) and indole-3-acetaldehyde oxidases (AAO1 and AAO2). *TIR1* encodes an auxin receptor that mediates auxin-regulated transcription. The observation of altered *TIR1* methylation levels implies that microgravity affects auxin signaling in plants.^28^ Aux/IAA proteins are short-lived TFs that function as repressors of early auxin response genes.^29^ Repression is thought to result from the interaction of the repressors with auxin response factors (ARFs). Formation of heterodimers with ARF proteins may alter the ability of these proteins to modulate the expression of genes involved in the early auxin response. The decreased methylation level of *ARF* genes and the increased methylation level of the *IAA9* and *IAA33* genes may contribute to an increased auxin response. The gravimorphogenesis of plant organs was changed due to the effects of microgravity on the polar transport of auxin.^30^ *BIG* encodes a membrane-associated protein and is required for auxin transport. We found that *BIG* was hypermethylated in space; thus, microgravity might inhibit auxin transport by increasing *BIG* methylation. Based on these results, we concluded that microgravity affects auxin-related processes at the levels of perception, signaling, transport and biosynthesis.^31^ The data imply that the effects of microgravity on auxin-related processes observed in previous spaceflight experiments may be related to changes in DNA methylation levels. In addition, altered methylation levels of the *EIN2* and *EIN3* genes, which play important roles in the ethylene signaling pathway, were detected. Alterations in the methylation of the ethylene responsive transcription factor genes *ERF11* and *ERF094* were also involved in the response to microgravity. Moreover, the methylation levels of the genes involved in ABA biosynthesis (*ABA3*) and brassinosteroid insensitive1 associated receptor kinase (BAK1) were also decreased.

Alterations in the methylation of genes related to cell wall were also identified. Hypergravity has been shown to upregulate cell wall rigidity in stems and roots.^32^ However, plant cell wall rigidity is lower under microgravity conditions than under ground conditions.^33^ The thickness of cell walls decreased in response to the microgravity stimulus. The space-grown plants also contained xyloglucan and 1,3,1,4-β-glucans with lower molecular masses resulting from increases in xyloglucan-degrading and 1,3,1,4-b-glucanase activities. These results show that increasing plant cell wall rigidity via modification of the metabolism of cell wall constituents is an important step in gravity resistance. The results shown in Table S5 indicate that numerous genes related to cell wall biosynthesis, such as the gene encoding the cellulose synthase catalytic subunit, callose synthase genes and cell wall expansion-related genes such as xyloglucan endotransglucosylase, beta-galactosidase and beta-glucosidase, displayed altered methylation levels.

### Growth of *Arabidopsis* seedlings under microgravity conditions

**Fig. 6:**
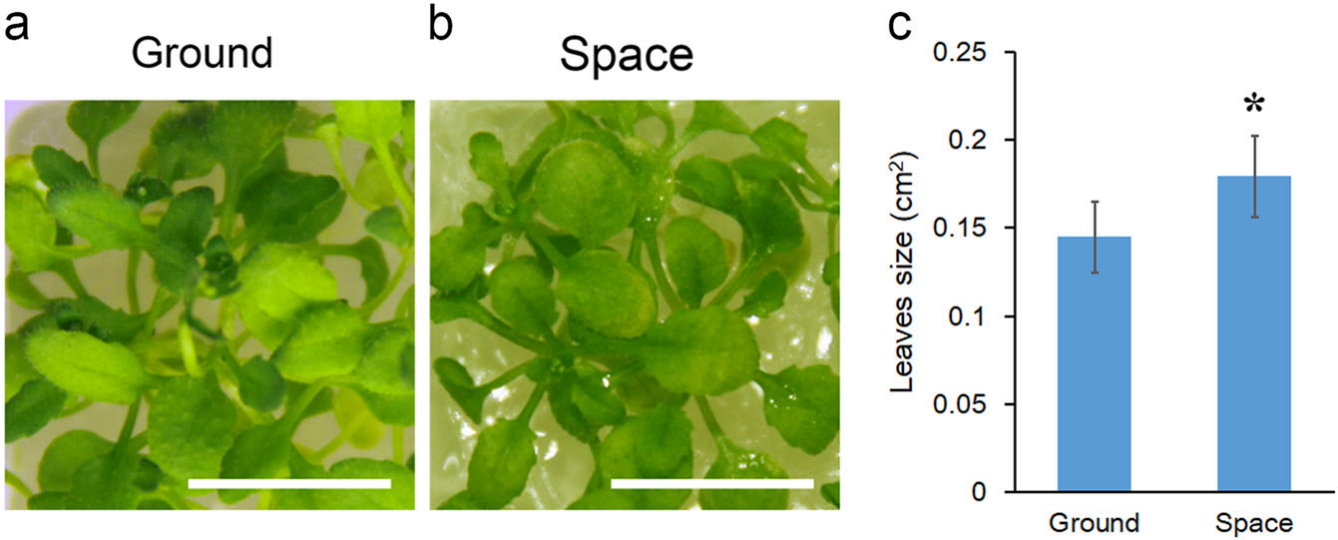
Comparison of the phenotypes of *Arabidopsis* seedlings grown under microgravity and on the ground. **a** Seedlings that served as ground control samples. **b** Seedlings that returned to earth with the satellite after growth under microgravity for 11 days. The experimental procedure was the same as described in Fig. 1d. The leaf area was measured using ImageJ. Bar = 1 cm (in **a**, **b**). The data shown are the mean values obtained for eight leaves (*n* = 8); the vertical bars represent the standard deviation. Asterisks indicate a statistically significant difference (*p* < 0.05) between spaceflight and control samples using Student’s two-tailed *t* test

The seedlings in the culture chamber installed in cultivation unit two grew for 11 days under microgravity conditions; at the end of the flight, they were alive and were recovered with the satellite. We obtained four *Arabidopsis* seedlings (Col-0) grown in space. These seedlings were photographed immediately on removal from the satellite and used for the measurement of leaf area. Their first and second true leaves were partially expanded at that time. We compared the areas of these eight true leaves with those of the leaves at the same leaf position of control *Arabidopsis* seedlings grown on the ground. The average leaf area of the seedlings grown under microgravity was 0.179 ± 0.023 cm², whereas that of the seedlings grown on the ground was 0.145 ± 0.020 cm². The results show that the leaves of the seedlings grown in microgravity were larger than those of the ground control samples (Fig. 6).

## Discussion

Plants are a crucial source of food, oxygen, and pharmaceuticals for people and animals, and they are a vital part of human life support systems for future long-duration spaceflight. However, space is not an ideal environment to grow plants, which are affected by microgravity and radiation. Global alterations in gene-expression patterns have been shown to occur in plants grown in space.^3,4^ Epigenetic modification of DNA is emerging as an important mechanism for modulating gene expression under different environmental conditions. The aim of this study was to examine the epigenetic mechanisms that regulate early seedling development in space under microgravity conditions. The results of this investigation can be used to improve strategies for designing plant strains that are better able to withstand microgravity and other adverse environmental conditions.

Epigenetic changes in response to spaceflight lead to altered cytosine DNA methylation in *Arabidopsis* seedlings grown under microgravity conditions. This research conducted a whole-genome bisulfate sequencing of *Arabidopsis* seedlings to discover and qualitatively assess spaceflight-associated 5-methyl cytosine (5mCyt) epigenetic modifications. The results demonstrated that lower methylation levels in the CHG, CHH, and CpG contexts in the *Arabidopsis* genome tended to occur under microgravity conditions (Fig. 2). However, this does not mean that all gene patterns were hypomethylated. As the results in Tables S1, S3, and S4 show, methylation-related genes (*AGO2*, *DME*, *DMT1*, and *APE1*), transcription factor genes (*MYB65, MYB74, BHLH35, BHLH81, BHLH131, BZIP68, GATA26, NAC081, NAC100, NAC105, WRKY7*, and *WRKY19*) and hormone-related genes (*ARF3, IAA9*, and *IAA3*3) were all hypermethylated. Some genes, including *MYB3R5, GATA14, ARF1, ARF2, ARF4, AAO2, BIG, BAK1, ABA*, and others, displayed more than two regions with opposite methylation patterns. The actual changes in the expression of these genes will require further experimental verification. Our results cannot be simply interpreted as indicating that the methylation of DNA is inhibited by microgravity, thereby contributing to hypomethylation of the *Arabidopsis* genome.

Previous research on the DNA methylome in *Solanum lycopersicum*, *Gossypium hirsutum* L., *Zea mays*, *Oryza sativa*, and *Glycine max*^13,34–37^ provides useful information for analyzing DNA methylation during *Arabidopsis* seedling development. Hypermethylation is usually found in centromeres and pericentromeric regions, and CpG methylation shows the highest levels among the various species.^38^ Our results consistently demonstrated that CHG, CHH, and CpG methylation patterns were related to TE density. These results show that DNA methylation plays an important role in the regulation of genome stability. In addition, DNA methylation has an important function in gene repression, although a recent report indicated that the DNA methylation pattern does not affect gene transcription in *Arabidopsis*.^39^ Compared to genetic variations, modifications in DNA methylation are more flexible. In response to environmental changes, epigenetic modifications enable plants to more easily adapt to new environments. Although some epigenetic modifications can be reversed, other epigenetic changes are heritable and can be passed on at least for one generation.^39,40^

In this report, analysis of the whole-genome methylome of *Arabidopsis* seedlings under microgravity conditions revealed numerous DMRs. The distribution and methylation levels of the DMRs of the five *Arabidopsis* chromosomes are shown in Figs. S3–S6. After analysis of the molecular functions of these DMRs, we found that approximately 36 TF genes showed altered methylation levels during microgravity spaceflight. It is proposed that the possible differentially methylated TF genes may contribute to microgravity adaptation in plants (Table S3). Furthermore, abundant DMRs were found to be involved in “Signaling” pathways, especially in hormone signaling pathways including auxin, ABA, ethylene, and BR (Table S4). Hormone-related genes whose expression was changed under microgravity conditions have also been identified in previous studies of the gene expression profiles of spaceflight specimens.^4^ Here, we were particularly concerned with AMR auxin-related genes. In fact, alterations in the methylation of auxin-related genes may play important roles in plants’ adaptation to microgravity. Auxin participates in plant gravitropism and is an important hormone that is involved in the relationship between plants and gravity. A previous spaceflight experiment in which GFP signaling was used to measure the expression of GFP reporter genes in pDR5r::GFP, pTAA1::TAA1–GFP, pSCR::SCR–transgenic *Arabidopsis* to monitor auxin did not detect an influence of microgravity on auxin distribution in the root tip region.^41^ However, in other spaceflight experiments involving plant cells and seedlings, auxin-related genes identified by transcriptomic analysis, including genes related to auxin metabolism, transport, perception, and signal transduction, showed changes in expression in the microgravity environment.^2,3^ The change in CsPIN1 distribution under microgravity also affects the local distribution of auxin.^30^ Therefore, the intensity and distribution of auxin in the cells of different parts of plant organs under microgravity conditions should be further characterized using more sensitive approaches.^42^ Moreover, signal transduction by auxin, as well as the mode and intensity of auxin’s action, may be affected by changes in the expression of auxin-related genes under microgravity conditions. Therefore, the information on changes in the DNA methylation of auxin-related genes presented in this paper contributes to the understanding of the changes in auxin effects and cell wall and plant phenotypes that occur under microgravity conditions.

Previous space experiments have shown that altered cell shape occurs as a result of cell wall perturbation under microgravity.^4^ This phenomenon may also be associated with changes in the expression of auxin-related genes under microgravity because the main effect of auxin is to relax the cell wall, thereby increasing cell growth. Growth induced by auxin is achieved by increasing the plasticity of the cell wall. During spaceflight, alterations in the methylation of cell wall-related genes were identified (Table S5). Changes in the expression of this category of genes in microgravity were also observed in several previous spaceflight experiments.^4^ Fig. 6 shows that the leaf growth of *Arabidopsis* seedlings was enhanced under microgravity. The most immediate effect that plants experience under space conditions is weightlessness. This is an environment that terrestrial plants have never encountered. Therefore, the most direct adaptive response of plants is the weakening of mechanical support tissue under microgravity.^43^ First, the rigidity of the plant cell wall decreases under microgravity conditions.^4,43^ As a result, the expression of cell wall genes is altered.^4^ Regulation of these gene-expression changes may be related to changes in the DNA methylation level in plant cells. We found changes in the DNA methylation levels of genes encoding transcription factors and certain genes associated with cell wall metabolism. Based on these results, the phenotypic acceleration of seedling growth under microgravity may be related to changes in the DNA methylome, in the expression of auxin-related genes and cell wall-related genes, and in cell wall rigidity. The results suggest that adjustment of cell wall rigidity by modification of the metabolism of the cell wall is an important mechanism through which plants adapt to changes in gravity. These inferences are supported by the results of previous space experiments in which it was demonstrated that increased plasticity of the cell wall enhanced the growth of the hypocotyl of *Arabidopsis*, rice coleoptile and flower stems of *Arabidopsis* under microgravity conditions.^43^

In addition to methylation alterations at the gene level, TEs are also involved in the adaptation of plants to environmental conditions via epigenetic variations.^22^ We demonstrated here that many TEs display reduced methylation levels under microgravity conditions. The relatively unstable DNA methylation of TEs plays an important role in the induction of active transposons.^36^ A global study of TEs under abiotic stress showed that many TEs show increased expression and that many of them function as enhancers of stress-induced genes in the maize genome.^44^ In addition, TE methylation levels may dynamically regulate transposon expression in pathogen-infected *Arabidopsis*.^24^ Coupled with our observations, this indicates that DNA demethylation within TEs may cause transcriptional alterations in transposons during adaptation to spaceflight conditions. Overall, our single-base resolution methylome research revealed novel microgravity-induced methylome alterations in *Arabidopsis* seedlings. These results are helpful for understanding the role of the regulation of methylation in response to microgravity conditions.

Because we used *Arabidopsis* seedlings from the beginning of the flight experiment, the possible effects from launch conditions on seedlings should also be considered. Therefore, to determine the effects of launch acceleration for 330 s on the DNA methylation pattern of *Arabidopsis* seedlings, we performed a simulated launch experiment on the ground; the results are shown in Supplementary Fig. S2. We measured changes in DNA methylation levels in specific regions of three genes. As the data show, there were no significant difference in *ABA3* and *DME*, but some hypermethylation was observed in *ARF1* (the methylation tendency of this gene under microgravity was reversed) after hypergravity treatment for 330 s. We, therefore, concluded that exposure to hypergravity for 330 s had little effect on DNA methylation in *Arabidopsis* seedlings that were subsequently exposed to microgravity for 60 h. Comparison of the results of preliminary hypergravity and microgravity experiments showed that the trend of DNA methylation changes of certain genes in plants grown under microgravity and hypergravity was reversed. The strength of this correlation should be tested by further extension of the hypergravity processing time and subsequent large-scale analysis. An opposite trend under hypergravity and microgravity experiments has previously been shown with respect to changes in intracellular calcium concentration.^45^ There is also a need for further study, whether there is a relationship between changes in gravity, changes in intracellular calcium concentration, and changes in DNA methylation. In addition, a 1-g centrifuge was not available due to space limitations within the satellite. To minimize errors, we extracted genomic DNA from one sample contained 49 *Arabidopsis* seedlings, and 3 independent samples were used in the analysis. However, we still cannot completely rule out the possibility that spaceflight disturbances other than microgravity effects affected the results obtained in our spaceflight experiments.

## Materials and methods

### Experimental setup

To meet the requirements for the SJ-10 spaceflight experiment, surface-sterilized seeds of *Arabidopsis thaliana* (Col-0) were sown in Petri dishes containing half-strength Murashige and Skoog medium and 1% phytagel and stratified at 4 °C for 3 days. After stratification, the Petri dishes containing the seeds were placed in a culture room with an ambient temperature of 22 ± 1 °C and 16 h light at 8000 lx. After germination and growth for 3 days in the Petri dishes, 49 seedlings were transferred to individual culture chambers in cultivation unit 1 and precultivated for 3 days until launch (Fig. 1c, cultivation unit 1). The seedlings continued to grow for up to 60 h under microgravity conditions. We fixed these seedlings with RNAlater® (Ambion, Foster City, CA, USA) under microgravity at 60 h of spaceflight. After the chemically fixed seedlings were returned to the ground, 49 seedlings in a culture chamber were transferred to a Falcon tube containing RNAlater® as a single sample. Three independent samples prepared in this manner were used for further DNA methylome analysis. The samples were preserved and stored at −80 °C before further processing for DNA isolation. The Falcon tubes containing the samples were transferred to −20 °C overnight and then thawed at 4 °C overnight. The samples were warmed to room temperature to dissolve the RNAlater® precipitate and used for DNA extraction. The seedlings, which were grown in cultivation unit 2 under microgravity for 11 days, were still growing when they returned to the ground. All the seedlings were maintained under 16 h of light at 8000 lx at 22 ± 1 °C during the spaceflight and on ground. The g-profile during launch of the SJ-10 satellite into orbit is described in the references.^46^ The g-profile during launch was as follows: the first-level maximum static overload was 4.8 g in flight for 150 s; the second-level maximum static overload was 6.0 g in flight for 180 s.

### Genome isolation and whole-genome bisulfite sequencing library construction

This protocol was based in part on a previously developed method.^47^ Total genomic DNA was isolated from *Arabidopsis* seedlings according to a previous protocol.^48^ Using a sonicator, 5 mg of genomic DNA was fragmented to a DNA length of approximately 200 bp. The DNA fragments were then treated three times using the *Qiagen* 59104 *EpiTect Bisulfite* Kit. λ DNA was used to determine the bisulfite conversion rate. Libraries were sequenced on the Illumina Hiseq2500 platform. Following library construction, sequencing of three independent spaceflight samples and three independent ground samples was performed (Fig. S1).

### Mapping of reads

Using the default parameters, alignment of bisulfite-treated reads to the *Arabidopsis* genome was conducted using Bismark software.^49^ Sequence reads were also transformed into fully bisulfite-converted versions before the reads were aligned to similarly converted versions of the genome. Methylation level analysis was conducted by the sliding-window approach, and all of the unmethylated or methylated reads were counted in the window.^50^ The relative proportions of CHG, CHH, and CpG contexts were then determined.

### DMR analysis

DMRs were counted using swDMR software with a sliding-window method (http://122.228.158.106/swDMR/). Significantly changed DMRs were detected by the Fisher test. DMR genes were analyzed. The identified genes were then analyzed for GO and KEGG (Kyoto Encyclopedia of Genes and Genomes) (http://www.genome.jp/kegg/) enrichment. KEGG analysis was used to determine the pathway enrichment of functional DMRs^46^ with *p* values less than 0.05.

### Analysis of TEs

TEs were isolated and analyzed using the Repeat Masker program (http://www.repeatmasker.org/). We further used Fisher’s exact test to investigate TEs with altered methylation levels. The false discovery rate method was used for multiple testing adjustments. *p* Values less than 0.05 was defined as methylated TEs with significant alterations.

### PCR program for bisulfite sequencing

The Genemark® Plant Genomic DNA Purification Kit was used to extract genomic DNA from samples pretreated with the QIAGEN® EpiTect Bisulfite Kit. rTaq from Takara, Inc., was used to clone fragments. The PCR program settings were as follows: 94 °C 3:00; 94 °C 0:10, 60 °C 1:30, 60 °C 3:00, 2 times; 94 °C 0:10, 59 °C 1:30, 60 °C 3:00, 2 times; 94 °C 0:10, 58 °C 1:30, 60 °C 3:00, 2 times; 94 °C 0:10, 57 °C 1:30, 60 °C 3:00, 32 times; and 60 °C 5:00. The primers used for *ARF1*, *ABA3*, and *DME* are shown in Table S6.

### Availability of data

The methylome data obtained in this study were deposited into the NCBI Short Read Archive (SRA); the SRA accession number is SRP134216.

## Electronic supplementary material

Supplemental material

## References

[1] Blancaflor, E. B. *Plant Gravitropism*. (Springer, New York, 2015).

[2] Correll MJ (2013). Transcriptome analyses of *Arabidopsis thaliana* seedlings grown in space: implications for gravity-responsive genes. Planta.

[3] Jin J, Chen HY, Cai WM (2015). Transcriptome analysis of *Oryza sativa* calli under microgravity. Microgravity Sci. Technol..

[4] Johnson CM, Subramanian A, Pattathil S, Correll MJ, Kiss JZ (2017). Comparative transcriptomics indicate changes in cell wall organization and stress response in seedlings during spaceflight. Am. J. Bot..

[5] Paul AL, Ferl RJ (2015). Spaceflight exploration in plant gravitational biology. Methods Mol. Biol..

[6] Mirouze M, Paszkowski J (2011). Epigenetic contribution to stress adaptation in plants. Curr. Opin. Plant Biol..

[7] Wang WS (2011). Drought-induced site-specific DNA methylation and its association with drought tolerance in rice (*Oryza sativa* L.). J. Exp. Bot..

[8] Zhu N (2015). The R2R3-type MYB gene O*sMYB91* has a function in coordinating plant growth and salt stress tolerance in rice. Plant Sci..

[9] Liang D (2014). Single-base-resolution methylomes of *Populus trichocarpa* reveal the association between DNA methylation and drought stress. BMC Genet.

[10] Sahu PP (2013). Epigenetic mechanisms of plant stress responses and adaptation. Plant Cell Rep..

[11] Steward N, Kusano T, Sano H (2000). Expression of *ZmMET1*, a gene encoding a DNA methyltransferase from maize, is associated not only with DNA replication in actively proliferating cells, but also with altered DNA methylation status in cold-stressed quiescent cells. Nucleic Acids Res.

[12] Vanyushin BF (2006). DNA methylation in plants. Curr. Top. Microbiol. Immunol..

[13] Cokus SJ (2008). Shotgun bisulphite sequencing of the *Arabidopsis* genome reveals DNA methylation patterning. Nature.

[14] Law JA (2010). A protein complex required for polymerase V transcripts and RNA-directed DNA methylation in *Arabidopsis*. Curr. Biol..

[15] Hu WR, Tang BC, Kang Q (2017). Progress of microgravity experimental satellite SJ-10. Aeron Aero Open Access J.

[16] WANG Y (2016). Establishing and evaluation of the microgravity level in the SJ-10 recoverable satellite. Aerospace China.

[17] Paul AL (2011). Parabolic flight induces changes in gene expression patterns in *Arabidopsis thaliana*. Astrobiology.

[18] Penterman J (2007). DNA demethylation in the Arabidopsis genome. Proc. Natl. Acad. Sci. USA.

[19] Paul AL (2012). Spaceflight transcriptomes: unique responses to a novel environment. Astrobiology.

[20] Briarty LG, Maher EP (2004). Reserve utilization in seeds of *Arabidopsis thaliana* germinating in microgravity. Int J. Plant Sci..

[21] Kriegs B, Theisen R, Schnabl H (2006). Inositol 1,4,5-trisphosphate and Ran expression during simulated and real microgravity. Protoplasma.

[22] Hashida SN (2006). The temperature-dependent change in methylation of the Antirrhinum transposon Tam3 is controlled by the activity of its transposase. Plant Cell.

[23] Xing MQ (2015). Global analysis reveals the crucial roles of DNA methylation during rice seed development. Plant Physiol..

[24] Dowen RH (2012). Widespread dynamic DNA methylation in response to biotic stress. Proc. Natl. Acad. Sci. USA.

[25] Kang J (2011). Plant ABC transporters. Arabidopsis Book.

[26] Zhao C, Avci U, Grant EH, Haigler CH, Beers EP (2008). XND1, a member of the NAC domain family in *Arabidopsis thaliana*, negatively regulates lignocellulose synthesis and programmed cell death in xylem. Plant J..

[27] Xie Q (2002). SINAT5 promotes ubiquitin-related degradation of NAC1 to attenuate auxin signals. Nature.

[28] Dharmasiri N, Dharmasiri S, Estelle M (2005). The F-box protein TIR1 is an auxin receptor. Nature.

[29] Goh T, Kasahara H, Mimura T, Kamiya Y, Fukaki H (2012). Multiple AUX/IAA-ARF modules regulate lateral root formation: the role of *Arabidopsis* SHY2/IAA3-mediated auxin signalling. Philos. Trans. R. Soc. B.

[30] Yamazaki C (2016). The gravity-induced re-localization of auxin efflux carrier CsPIN1 in cucumber seedlings: spaceflight experiments for immunohistochemical microscopy. NPJ Microgravity.

[31] Yamaguchi N, Komeda Y (2013). The role of CORYMBOSA1/BIG and auxin in the growth of *Arabidopsis* pedicel and internode. Plant Sci..

[32] Martzivanou M, Hampp R (2003). Hypergravity effects on the *Arabidopsis* transcriptome. Physiol. Plant.

[33] Hoson T (2014). Plant growth and morphogenesis under different gravity conditions: relevance to plant life in space. Life (Basel).

[34] Li Y (2017). Genome-wide comparative analysis of DNA methylation between soybean cytoplasmic male-sterile line NJCMS5A and its maintainer NJCMS5B. BMC Genom..

[35] Lu X (2017). Single-base resolution methylomes of upland cotton (*Gossypium hirsutum* L.) reveal epigenome modifications in response to drought stress. BMC Genom..

[36] Zemach A (2010). Local DNA hypomethylation activates genes in rice endosperm. Proc. Natl. Acad. Sci. USA.

[37] Zhong S (2013). Single-base resolution methylomes of tomato fruit development reveal epigenome modifications associated with ripening. Nat. Biotechnol..

[38] Lister R (2008). Highly integrated single-base resolution maps of the epigenome in *Arabidopsis*. Cell.

[39] Meng D (2016). Limited contribution of DNA methylation variation to expression regulation in *Arabidopsis thaliana*. PLoS Genet.

[40] Bird A (2002). DNA methylation patterns and epigenetic memory. Genes Dev..

[41] Ferl RJ, Paul AL (2016). The effect of spaceflight on the gravity-sensing auxin gradient of roots: GFP reporter gene microscopy on orbit. NPJ Microgravity.

[42] Liao CY (2015). Reporters for sensitive and quantitative measurement of auxin response. Nat. Methods.

[43] Hoson T (2014). Growth stimulation in inflorescences of an *Arabidopsis* tubulin mutant under microgravity conditions in space. Plant Biol. (Stuttg.).

[44] Makarevitch I (2015). Transposable elements contribute to activation of maize genes in response to abiotic stress. PLoS Genet.

[45] Hausmann N (2014). Cytosolic calcium, hydrogen peroxide and related gene expression and protein modulation in *Arabidopsis thaliana* cell cultures respond immediately to altered gravitation: parabolic flight data. Plant Biol. (Stuttg.).

[46] Mao X, Cai T, Olyarchuk JG, Wei L (2005). Automated genome annotation and pathway identification using the KEGG Orthology (KO) as a controlled vocabulary. Bioinformatics.

[47] Rackham OJ, Dellaportas P, Petretto E, Bottolo L (2015). WGBSSuite: simulating whole-genome bisulphite sequencing data and benchmarking differential DNA methylation analysis tools. Bioinformatics.

[48] Irfan M (2013). Modification of CTAB protocol for maize genomic DNA extraction. Res. J. Biotechnol..

[49] Krueger F, Andrews SR (2011). Bismark: a flexible aligner and methylation caller for Bisulfite-Seq applications. Bioinformatics.

[50] Lister R (2013). Global epigenomic reconfiguration during mammalian brain development. Science.

