# Supplementary material for: Single-base resolution methylome analysis shows epigenetic changes in Arabidopsis seedlings exposed to microgravity spaceflight conditions on board the SJ-10 recoverable satellite
Source: NPJ Microgravity. 2018 Jul 12;4:12. doi: 10.1038/s41526-018-0046-z (PMC6043569; doi:10.1038/s41526-018-0046-z)
Supplement: Supplementary file 1 — Supplemental material [file 41526_2018_46_MOESM1_ESM.pdf]

Supplementary data

Article title: **Single-base Resolution Methylome Analysis Shows Epigenetic Changes in *Arabidopsis* Seedlings Exposed to Microgravity Spaceflight Conditions on Board the SJ-10 Recoverable Satellite**

Peipei Xu[1#], Haiying Chen[1#], Jing Jin[1], Weiming Cai[1*]

[1]Laboratory of Photosynthesis and Environment, CAS Center for Excellence in Molecular Plant Sciences, Shanghai Institute of Plant Physiology and Ecology , Chinese Academy of Sciences, No. 300 Fenglin Road, Shanghai 200032, China

Figure S1:

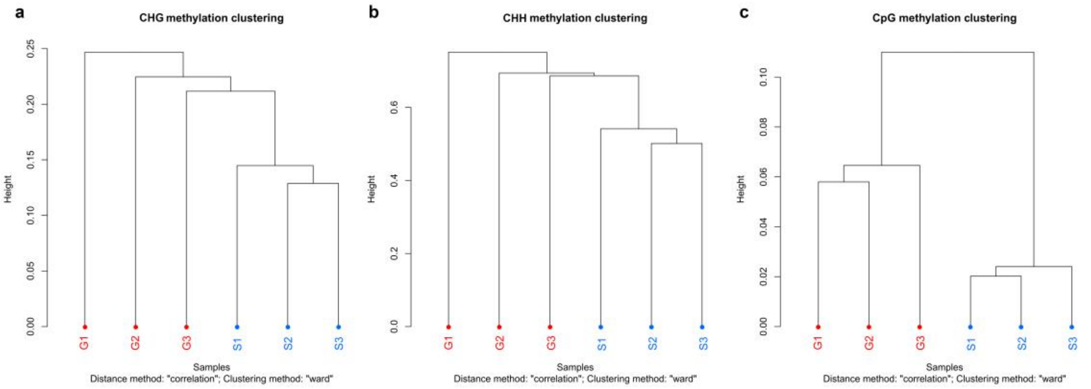

Cluster analysis of three on-ground samples (G1, G2, G3) and three independent space samples (S1, S2, S3) of the CHG (a), CHH (b) and CpG(c) contexts.

Figure S2:

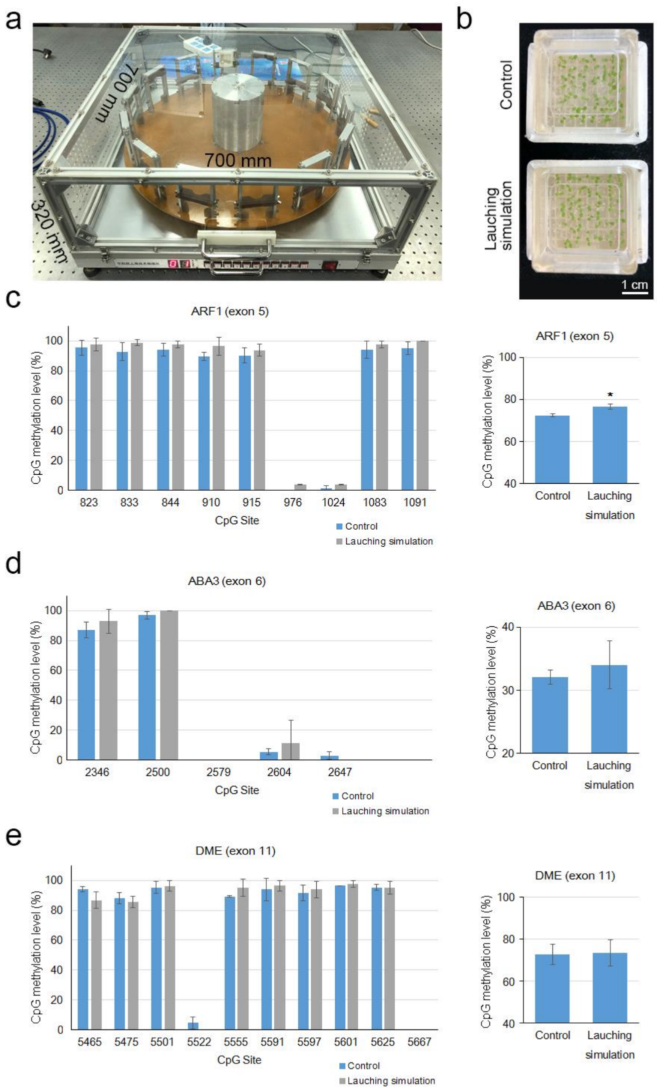

Study on biological effects of simulated satellite launching in *Arabidopsis*.

(a) Centrifuge used in hypergravity experiment and (b) the phenotype of the seedlings after hypergravity experiment. (c-e) Bisulfite sequencing analysis of methylation levels in exon regions of *ARF1* (823 - 1091)，*ABA3* (2346 - 2647) and *DME* (5466 - 5667), n = 3, *: $p < 0.05$.

The experimental procedure and conditions of cultivation of *Arabidopsis* seedlings were same as described in Fig. 1d (before launching time). After 330 seconds of hypergravity treatment, the seedlings were used immediately for DNA extraction. Launching simulation: the first-level overload was 5.0 g for 150 seconds; the second-level overload was 6.0 g for 180 seconds. The changes of methylation level of the exon region of these three genes under microgravity were shown in Table S1 and Table S4.

Figure S3:

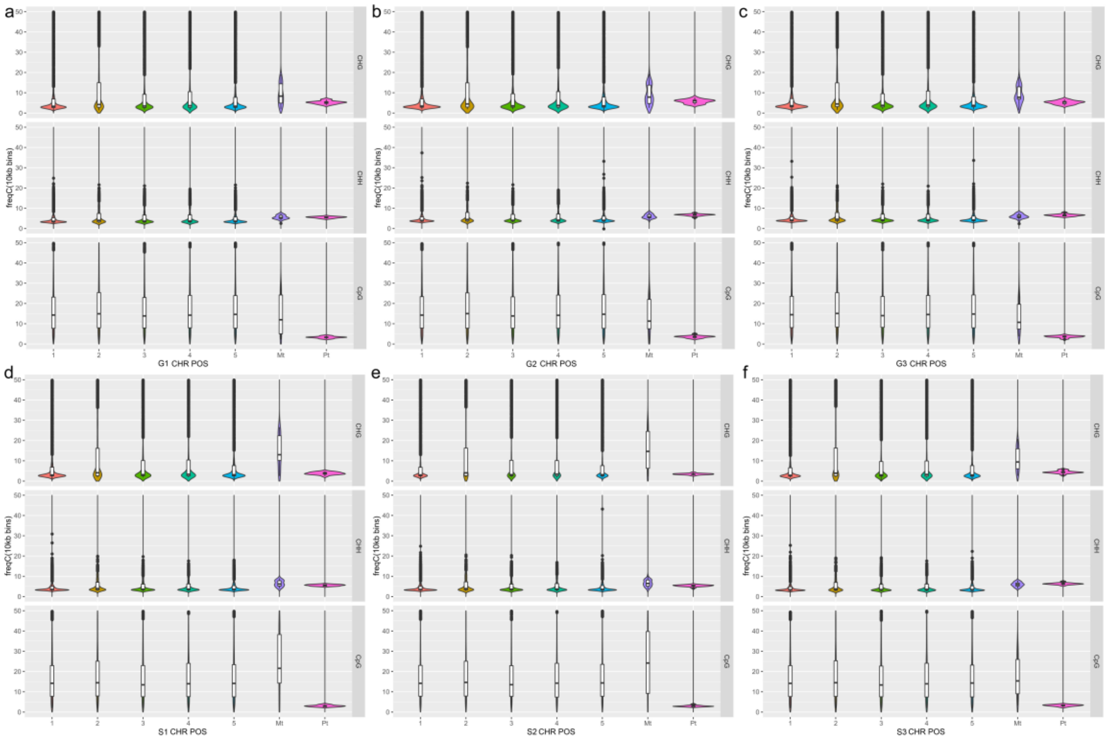

CpG methylcytosine distribution of the three independent space sample S1(a), sample S2(b), sample S3(c) and three on ground sample G1(d), sample G2(e), sample G3(f) of CpG, CHH and CHG context on five chromosomes and Mt, Pt.

Figure S4:

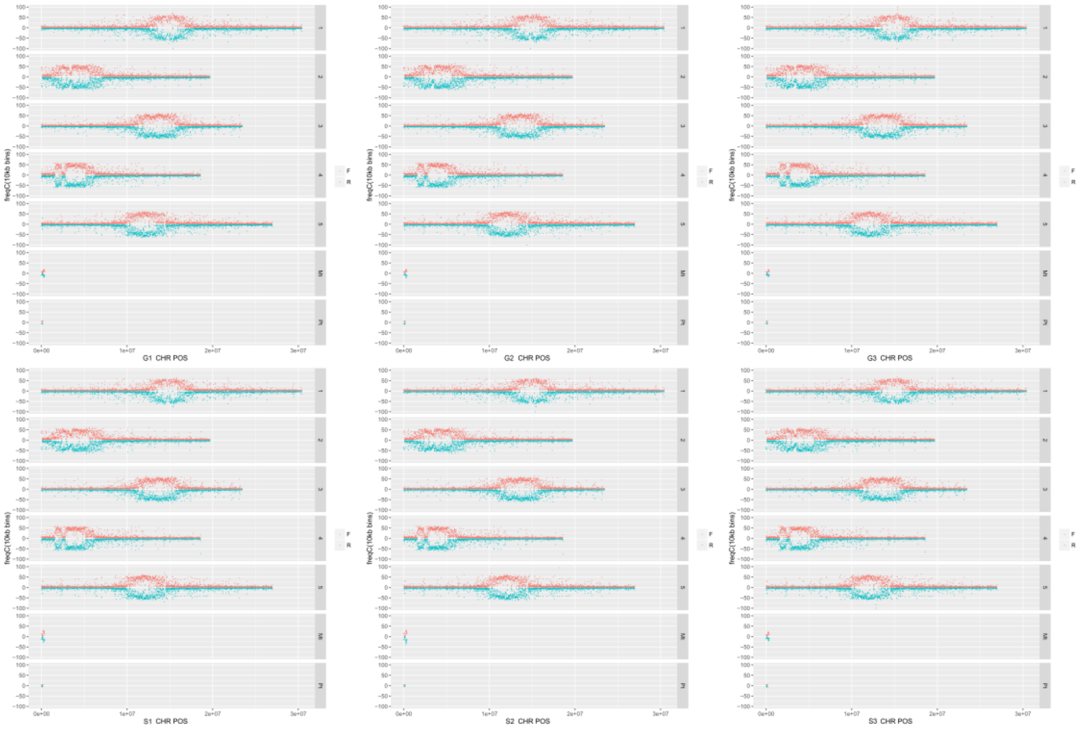

CHG methylcytosine distribution of the three independent space samples (S1,S2,S3) and three on ground samples (G1,G2,G3) on 5 chromosomes and Mt, Pt.

Figure S5:

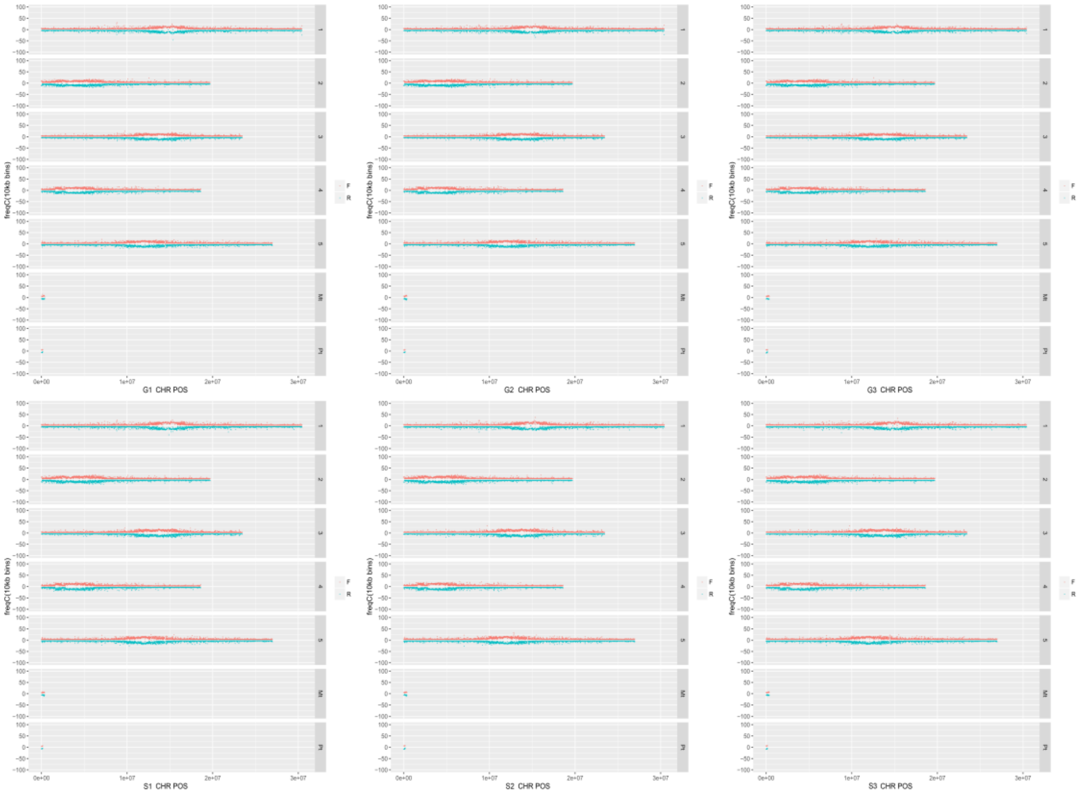

CHH methylcytosine distribution of the three independent space samples (S1,S2,S3) and three on ground samples (G1,G2,G3) on 5 chromosomes and Mt, Pt.

Figure S6:

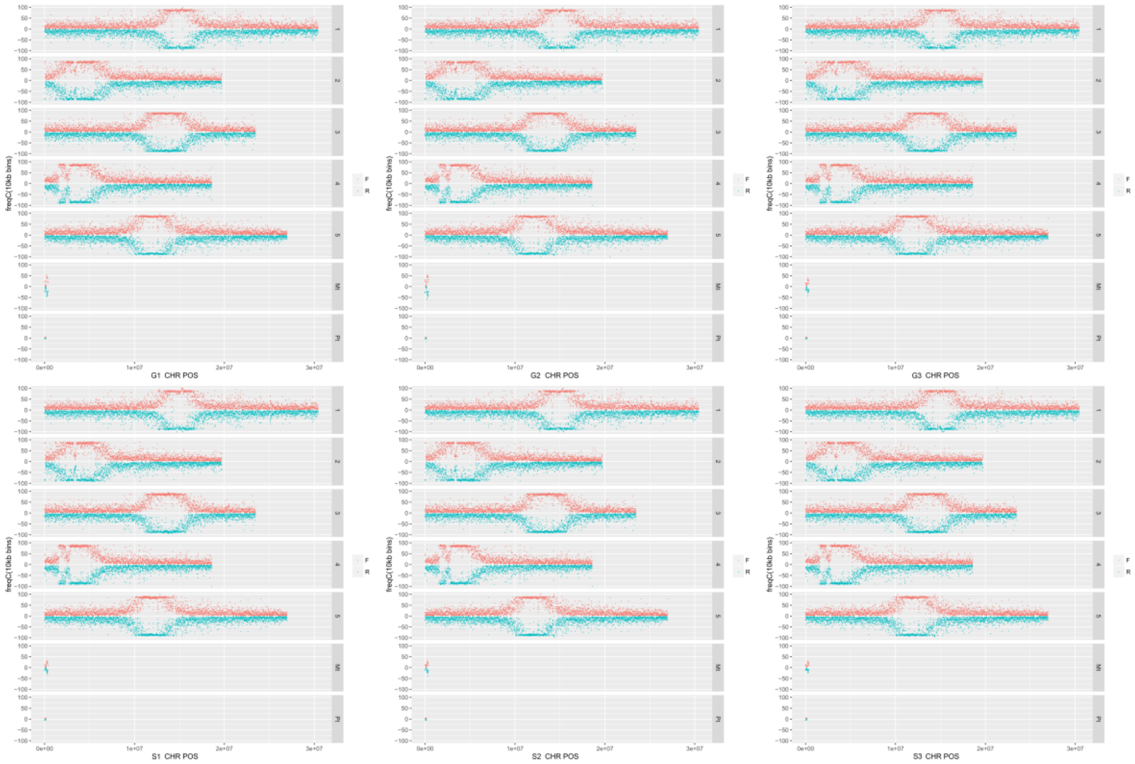

Distributions of CpG methylcytosine density of all the six samples on 5 chromosomes and Mt, Pt.

Table S1. Methylation-related genes which showed altered methylation levels in *Arabidopsis* grown in microgravity compared with the control grown under 1g ($p < 0.05$).

Gene name	Locus	Annotation	Diff. Methy (S-G)
Protein argonaute			
AGO2	AT1G31280	exon 3 of 3	0.213
		exon 3 of 3	0.173315
AGO8	AT5G21030	exon 5 of 21	-0.29709
		exon 6 of 21	-0.36424
AGO10	AT5G43810	exon 20 of 20	-0.2002
		promoter-TSS	-0.22464
		TTS	-0.37014
DNA (cytosine-5)-methyltransferase			
CMT3	AT1G69770	exon 7 of 21	-0.36427
S-adenosyl-L-methionine-dependent methyltransferases superfamily protein			
DRM3	AT3G17310	intron 6 of 9	-0.35597
SNF2 domain-containing protein			
CLSY1	AT3G42670	TTS	-0.20012
CLSY2	AT5G20420	TTS	0.190565
CLSY4	AT3G24340	exon 3 of 3	-0.28239
Histone-lysine N-methyltransferase			
SUVH3	AT1G73100	TTS	-0.37213
SUVH4	AT5G13960	intron 12 of 14	-0.25522
SUVH5	AT2G35160	exon 3 of 3	-0.39782
		exon 3 of 3	-0.57018
		exon 3 of 3	-0.40895
SUVH6	AT2G22740	exon 2 of 2	-0.4596
RNA-binding (RRM/RBD/RNP motifs) family protein			
ROS3	AT5G58130	exon 4 of 4	-0.29129
Transcriptional activator			
DME	AT5G04560	exon 11 of 18	0.182532
DNA (cytosine-5)-methyltransferas			
DMT1	AT5G49160	exon 2 of 12	0.286685
Acclimation of photosynthesis to environment			
APE1	AT5G38660	intron 5 of 12	0.333855
Tetraspanin			
TET14	AT2G01960	TTS	-0.31082

Table S2. Data description of the BS-Seq reads for the ground controls (G1, G2, G3) and microgravity (S1, S2, S3) samples.

Sample Name	Total Reads	Total Base	Clean Reads	Clean Base	Clean Q20	Clean Q30	Clean Ratio (%)
G1	48,909,868	7,385,390,068	45,163,828	5,761,781,276	0.9834	0.9584	92.34
G2	77,033,546	11,632,065,446	69,186,664	8,638,159,382	0.9834	0.9583	89.81
G3	59,075,149	8,920,347,499	53,997,002	6,657,628,817	0.9834	0.9586	91.40
S1	35,199,598	5,279,939,700	35,016,558	5,120,635,215	0.9748	0.9378	99.48
S2	35,145,327	5,271,799,050	34,947,415	5,102,302,329	0.9742	0.9366	99.44
S3	39,029,346	5,869,115,553	38,884,399	5,726,862,538	0.9784	0.9462	99.63

Table S3. Transcription factor genes that showed altered methylation level in *Arabidopsis* grown in microgravity (p < 0.05).

Gene name	Locus	Annotation	Diff. Methy (S-G)
ATMYB35	AT3G28470	promoter-TSS	-0.393009
ATMYB3R5	AT5G02320	TTS	0.363732
		exon 8 of 9	-0.377741
ATMYB65	AT3G11440	exon 3 of 4	0.548274
ATMYB74	AT4G05100	promoter-TSS	0.401997
BHLH28	AT5G46830	promoter-TSS	-0.145863
BHLH35	AT5G57150	TTS	0.220791
BHLH41	AT5G56960	TTS	-0.153077
BHLH66	AT2G24260	TTS	-0.435487
BHLH81	AT4G09180	intron 3 of 4	0.394391
BHLH95	AT1G49770	promoter-TSS	-0.204263
BHLH123	AT3G20640	TTS	-0.319919
BHLH131	AT4G38070	TTS	0.296181
BHLH140	AT5G01310	exon 3 of 6	-0.189424
BHLH144	AT1G29950	exon 4 of 5	-0.316130
BZIP16	AT2G35530	exon 5 of 13	-0.515903
BZIP28	AT3G10800	TTS	-0.489383
BZIP30	AT2G21230	TTS	-0.36030
BZIP63	AT5G28770	promoter-TSS	-0.318241
BZIP68	AT1G32150	exon 9 of 13	0.263631
GATA	AT3G25660	intron 3 of 8	0.186431
GATA10	AT1G08000	TTS	0.229709
GATA14	AT3G45170	exon 2 of 2	-0.200013
	AT3G45170	TTS	0.170634
GATA26	AT4G17570	exon 3 of 8	0.281708
NAC008	AT1G25580	exon 4 of 6	-0.348574
NAC023	AT1G60280	TTS	-0.236220
NAC039	AT2G24430	TTS	-0.236220
NAC043	AT2G46770	promoter-TSS	-0.236220
NAC069	AT4G01550	promoter-TSS	-0.236220
NAC081	AT5G08790	promoter-TSS	0.268681
NAC100	AT5G61430	promoter-TSS	0.268681
NAC105	AT5G66300	promoter-TSS	0.268681
		exon 1 of 3	0.268681
WRKY1	AT2G04880	TTS	-0.408020
WRKY7	AT4G24240	intron 11 of 14	0.292929
WRKY19	AT4G12020	intron 4 of 5	0.270506
WRKY20	AT4G26640	exon 3 of 6	-0.499226
	AT4G26640	promoter-TSS	-0.548767
WRKY74	AT5G28650	promoter-TSS	-0.434246

Table S4. Hormone related genes that showed altered methylation level in *Arabidopsis* grown in microgravity ($p < 0.05$).

Gene name	Locus	Annotation	Diff. Methy (S-G)
Auxin response factor			
ARF1	AT1G59750	exon 5 of 15	-0.50845
		exon 6 of 15	-0.53716
		exon 13 of 15	0.260059
ARF2	AT5G62000	exon 12 of 16	-0.51619
		exon 13 of 16	-0.65464
		exon 15 of 16	0.26779
ARF3	AT2G33860	intron 5 of 9	0.30883
ARF4	AT5G60450	exon 10 of 12	-0.34222
		exon 10 of 12	-0.38993
		intron 5 of 11	0.317819
ARF5	AT1G19850	TTS	-0.32076
ARF6	AT1G30330	exon 13 of 14	-0.48895
ARF6	AT1G30330	exon 14 of 14	-0.28162
ARF8	AT5G37020	intron 4 of 13	-0.25235
ARF19	AT1G19220	exon 8 of 12	-0.32607
ARF21	AT1G34410	intron 3 of 13	-0.36508
ARF22	AT1G34390	exon 3 of 14	-0.130253
Auxin signaling F-box			
AFB2	AT3G26810	exon 3 of 3	0.26723
AFB5	AT5G49980	exon 2 of 3	-0.20794
TIR	AT1G72930	TTS	0.190826
Auxin-responsive protein			
IAA9	AT5G65670	exon 3 of 6	0.204658
		intron 2 of 5	0.18341
IAA33	AT5G57420	TTS	0.580797
Indole-3-acetaldehyde oxidase			
AAO1	AT5G20960	exon 2 of 10	0.237555
AAO2	AT3G43600	exon 2 of 9	-0.49537
		exon 2 of 9	0.535264
		exon 5 of 9	-0.32953
Auxin transport protein			
BIG	AT3G02260	exon 4 of 14	0.230049
		exon 4 of 14	0.281997
		exon 4 of 14	0.279249
		exon 8 of 14	-0.52414
		exon 12 of 14	0.283206
Brassinosteroid insensitive 1-associated receptor kinase			
BAK1	AT4G33430	intron 9 of 10	-0.40569
		exon 10 of 12	0.187463

ABA deficient			
ABA3	AT1G16540	promoter-TSS	0.211757
		promoter-TSS	-0.30194
		exon 6 of 21	-0.3429
		exon 9 of 21	0.263019
Ethylene-insensitive protein			
EIN2	AT5G03280	exon 7 of 8	0.303509
EIN3	AT3G20770	exon 2 of 2	-0.42091
		exon 2 of 2	0.048629
Ethylene-responsive transcription factor			
ERF11	AT1G28370	TTS	-0.36503
		TTS	-0.30229
ERF094	AT1G06160	promoter-TSS	0.257507
Ethylene response sensor			
ERS1	AT2G40940	TTS	-0.31638

Table S5. Cell wall related genes that showed altered methylation level in *Arabidopsis* grown in microgravity compared with that grown under 1*g* control ($p < 0.05$).

Gene name	Locus	Annotation	Diff. Methy (S-G)
Root meristem growth factor			
RGF3	AT2G04025	TTS	0.210603
RGF4	AT3G30350	promoter-TSS	0.273889
Cellulose synthase A catalytic subunit			
CESA1	AT4G32410	exon 10 of 14	-0.41712
CESA5	AT5G09870	exon 5 of 13	-0.321
CESA6	AT5G64740	exon 10 of 13	-0.20271
		exon 13 of 13	0.192504
CESA8	AT4G18780	exon 6 of 12	-0.33978
		exon 6 of 12	-0.25971
CESA9	AT2G21770	intron 5 of 12	-0.37932
Callose synthase			
CALS1	AT1G05570	exon 41 of 44	-0.41977
CALS3	AT5G13000	exon 33 of 44	0.20095
		exon 40 of 44	-0.35827
		exon 43 of 44	-0.38991
		intron 22 of 42	-0.23397
		intron 26 of 42	-0.45092
CALS6	AT3G59100	intron 16 of 41	-0.3966
CALS7	AT1G06490	exon 23 of 42	0.348981
		intron 19 of 41	-0.27468
CALS8	AT3G14570	exon 27 of 42	-0.33138
CALS9	AT3G07160	exon 3 of 50	-0.44072
		exon 35 of 50	0.201205
		intron 35 of 49	0.059917
		intron 41 of 49	-0.38041
CALS10	AT2G36850	intron 5 of 49	-0.35113
		intron 34 of 49	0.378681
		intron 44 of 49	-0.44741
CALS12	AT4G03550	exon 2 of 5	-0.22841
		exon 2 of 5	0.306824
		intron 2 of 4	0.34053
Xyloglucan endotransglucosylase / hydrolase			
XTH10	AT2G14620	exon 4 of 4	0.323687
XTH17	AT1G65310	promoter-TSS	0.24495
Pectinesterase / pectinesterase inhibitor			
PME14	AT2G36700	exon 3 of 5	0.280889
PME19	AT1G11590	TTS	-0.32367
		exon 2 of 3	0.245865
PME42	AT4G03930	promoter-TSS	-0.19401

PME48	AT5G07410	TTS	0.246784
PME49	AT5G07420	promoter-TSS	0.206408
PME51	AT5G09760	TTS	-0.34578
Starch synthase			
SS1	AT5G24300	exon 2 of 16	-0.1843
SS2	AT3G01180	exon 8 of 8	0.219161
SS4	AT4G18240	exon 12 of 16	-0.39843
		TTS	0.18302
Beta-galactosidase			
BGAL5	AT1G45130	intron 11 of 16	0.265369
BGAL9	AT2G32810	TTS	0.311635
BGAL12	AT4G26140	intron 15 of 16	0.527798
BGAL15	AT1G31740	promoter-TSS	-0.20524
BGAL16	AT1G77410	exon 15 of 19	-0.26202
		TTS	-0.21557
Beta-glucosidase			
BGLU24	AT5G28510	TTS	0.380285
BGLU42	AT5G36890	promoter-TSS	0.130545
BGLU45	AT1G61810	TTS	-0.16838

Table S6. Primers used for bisulfite sequencing analysis of methylation level.

ARF1 F	TTTTGTTGTGTTTTATTTTGTAAGT
ARF1 R	AAAAACAATCATCTACATATCTCC
ABA3 F	TTAGTTTTTGATATGATTTTTTT
ABA3 R	TTAAAAATTAAAAACCCTACAC
DME F	TTAGAGGAGAGGAGTTTAATAAGTG
DME R	AAAATCATTCCAAACTATTCAATAT
